# The systemic influence of platelet-derived growth factors on bone marrow mesenchymal stem cells in fracture patients

**DOI:** 10.1186/s12916-014-0202-6

**Published:** 2015-01-13

**Authors:** Hiang Boon Tan, Peter V Giannoudis, Sally A Boxall, Dennis McGonagle, Elena Jones

**Affiliations:** NIHR Leeds Biomedical Research Unit, Chapel Allerton Hospital, Leeds West Yorkshire, Leeds, LS7 4SA UK; Leeds Institute of Rheumatic & Musculoskeletal Medicine, University of Leeds, Room 5.24, Clinical Sciences Building, Leeds, LS9 7TF UK; Academic Unit of Trauma and Orthopaedics, Leeds General Infirmary, Great George St, Leeds, LS1 3EX UK

**Keywords:** Mesenchymal stem cells, MSCs, Bone marrow, PDGF, Platelets

## Abstract

**Background:**

Fracture healing is a complex process regulated by a variety of cells and signalling molecules which act both locally and systemically. The aim of this study was to investigate potential changes in patients’ mesenchymal stem cells (MSCs) in the iliac crest (IC) bone marrow (BM) and in peripheral blood (PB) in relation to the severity of trauma and to correlate them with systemic changes reflective of inflammatory and platelet responses following fracture.

**Methods:**

ICBM samples were aspirated from two trauma groups: isolated trauma and polytrauma (n = 8 and 18, respectively) at two time-points post-fracture and from non-trauma controls (n = 7). Matched PB was collected every other day for a minimum of 14 days. BM MSCs were enumerated using colony forming-fibroblast (CFU-F) assay and flow cytometry for the CD45-CD271+ phenotype.

**Results:**

Regardless of the severity of trauma, no significant increase or decrease in BM MSCs was observed following fracture and MSCs were not mobilised into PB. However, direct positive correlations were observed between changes in the numbers of aspirated BM MSCs and time-matched changes in their serum PDGF-AA and -BB. *In vitro*, patients’ serum induced MSC proliferation in a manner reflecting changes in PDGFs. PDGF receptors CD140a and CD140b were expressed on native CD45-CD271+ BM MSCs (average 12% and 64%, respectively) and changed over time in direct relationship with platelets/PDGFs.

**Conclusions:**

Platelet lysates and other platelet-derived products are used to expand MSCs *ex vivo*. This study demonstrates that endogenous PDGFs can influence MSC responses *in vivo*. This indicates a highly dynamic, rather than static, MSC nature in humans.

**Electronic supplementary material:**

The online version of this article (doi:10.1186/s12916-014-0202-6) contains supplementary material, which is available to authorized users.

## Background

Fracture healing is a highly co-ordinated physiological process with initial haematoma formation followed by an acute inflammatory response leading to an osteogenic repair phase, all tightly regulated by a variety of cytokines and other mediators and cells [1]. The systemic inflammatory response associated with fracture can be indirectly ascertained by elevation of C-reactive protein (CRP) and total white cell counts as well as up-regulation in several inflammation-related cytokines and chemokines [2-5]. The later osteogenic phase is mediated locally via the recruitment of mesenchymal stem cells (MSCs) from adjacent periosteum, cortical bone and bone marrow [6].

Given the systemic nature of fracture repair, the question arises whether MSCs at remote sites may undergo changes in their local environment, even to the degree that such MSCs may enter the systemic circulation. The concept of systemic recruitment and homing of human MSCs following fracture remains controversial but evidence for this exists in rodent models [7,8]. In humans, MSC-like cells have been reported in the peripheral circulation of burn victims [9], following stroke [10] or muscular damage [11] but not in any measurable numbers in healthy controls [12,13]. Therefore, it is possible that following fracture, and potentially in relation to the severity of fracture, certain alterations in MSCs at remote sites away from fracture, including alterations in surface receptor expression or even more profound changes sufficient to dislodge MSCs into the systemic circulation, may occur.

Indeed several molecules associated with the systemic inflammatory fracture repair response, such as platelet-derived growth factors (PDGFs) [14], have been shown to recruit MSCs by chemoattraction [15]. Fracture is known to up-regulate the production of PDGF-BB [16] and a positive effect of PDGF-BB on MSC proliferation and migration *in vitro* is well-documented [14,17-19]. Therefore, we hypothesised that powerful signalling molecules, such as the PDGFs released into circulation after fracture, may affect MSCs at sites remote to the initial injury. To address this hypothesis we investigated MSCs in the iliac crest (IC) bone marrow (BM) aspirates and peripheral blood (PB) of patients suffering differing severities of injury (fractures) and correlated MSC changes with patient- and time-matched changes in serum PDGFs as well as total white cell and platelet counts. MSCs were enumerated using a classical colony-forming assay-fibroblast (CFU-F) and flow cytometry for the CD45-CD271+ phenotype [20-22].

Herein, we report numerical and receptor expression changes in MSCs post-fracture in the IC BM, a site remote from fracture that correlated with time-matched changes in serum PDGFs. However, even the most severe injury was not sufficient to mobilize MSCs into the PB. These data provide the first evidence of the systemic effect of endogenous platelet-derived growth factors on the *in vivo* MSC pool in humans.

## Methods

### Patient recruitment

Ethical approval was obtained for the study (reference 06/Q1206/127, Leeds (East) REC), with all patients providing written informed consent. To address the influence and role of differing severities of trauma on MSC dynamics, patients were recruited into two groups based on the severity of injury as defined by their Injury Severity Score (ISS) [23]. Briefly, patients in the isolated trauma group had ISS <16 and patients in the polytrauma group had ISS ≥16. A third group of patients with no acute trauma or injuries who were undergoing elective orthopaedic surgery formed the control group. The demographics of the recruited patients are summarised in Table 1. For MSC proliferation experiments using the patient’s sera, cultured MSCs from four additional control subjects (two male/two female, median age 31, range 21 to 35), characterised in our previous study [24], were utilized.

**Table 1:** **Patient demographics (total number =33)**

**Patient group**	**Isolated trauma**	**Polytrauma**	**Control** ^**a**^
**Number of patients**	**n = 8**	**n = 18**	**n = 7**
Sex	7 M/1 F	13 M/5 F	3 M/4 F
Median age (range)	39 (21 to 64) years	39 (21 to 54) years	40 (19 to 52) years
Median ISS (range)	6 (4 to 9)	27 (22 to 50)^b^	n/a
Mechanism of injury	4 RTA, 4 fall from height	14 RTA, 3 fall from height, 1 assault	n/a
Median follow-up period (range)	124 (22 to 160) weeks	105 (31 to 158) weeks	n/a

^a^Control group were patients admitted electively for removal of metalwork; ^b^ *P* <0.05. F, female; ISS, injury severity score; M, male; RTA, road traffic accident.

### Sample collection

Patients recruited into both trauma groups had collection of IC BM as well as PB. IC BM aspiration could only be carried out when the patient was undergoing surgery, with the initial sampling occurring within 24 hours of injury (acting as baseline, day 0) and the subsequent sampling, dictated by clinical need for a further intervention, several days later (range 3 to 32 days). In addition, four patients had a third sampling time point occurring over a year post-trauma (range 397 to 602 days), when they were admitted for elective (non-acute trauma) operations.

For BM aspiration, consistency was assured in terms of surgeon (same person for all patient samples), aspirate location (anterior iliac crest, approximately 5 to 6 cm posterior to the anterior superior iliac spine), tools (Stryker 306–111, 11-gauge, bevel tipped trocar, Kalamazoo, MI, USA), volume of aspirate (20 ml) and draw method (single draw to fill full 20 ml syringe [25]). The aspirate was collected into an ethylenediaminetetraacetic acid (EDTA)-containing tube (BD Vacutainer, Oxford, UK). PB was collected every other day for 14 days and also time-matched to each BM aspiration episode; samples were taken into three tubes for different investigations, with 20 ml collected in EDTA for the MSC enumeration study, 20 ml taken in a clot accelerant tube (BD Vacutainer, Oxford, UK) for serum isolation, and the final sample (5 ml) sent to the hospital haematology laboratory for processing of full blood count, including platelet count and CRP.

### Sample processing and CFU-F assay

For MSC enumeration in both the IC BM aspirate and PB, samples were initially processed for mononuclear cell (MNC) isolation [20]. In brief, the sample was diluted 1:1 with phosphate buffered saline (PBS, Invitrogen, Paisley UK), before being carefully layered onto Lymphoprep (Axis-Shield, Dundee, UK) and subsequent centrifugation at 650 g for 25 minutes. The MNC fraction was washed twice in PBS and seeded into 10-cm diameter dishes (Corning, Tewksbury, MA, USA) in 10 ml of MSC culture media (NH Media, Milteny-Biotec, Bisley, UK), at 3 × 10^6^ MNCs/dish, in triplicate, for the CFU-F assay. The remaining MNCs were frozen down in freezing media and stored in liquid nitrogen for flow cytometry investigations. Expecting much lower MSC frequency in PB [12], PB MNCs were seeded at a higher density (10 × 10^6^ MNC/dish). CFU-F cultures were maintained for two weeks and CFU-F colonies scored, as described previously [26]. The technical variation (between the three dishes) in colony counts was approximately 23%. For all patients/data points, the CFU-F data are presented as the number of CFU-F/ml, calculated according to the formula:

$$ CFU-F/ ml = \left( mean\  of\  triplicate\ CFU-F/ dish/3\right) \times MNC\  count/ ml $$

### MSC enumeration using flow cytometry

Bone marrow MNCs were defrosted and the MSC frequency was measured based on the CD45-CD271+ phenotype, as previously described [24], with some modifications. Bone marrow MNCs were re-suspended at 1 x 10^7^ cells/ml in FACs buffer (PBS +0.5% bovine serum albumin (BSA) +2 mM EDTA). Antibodies were added at the manufacturers’ recommended concentrations and the cells were incubated with the antibodies for 20 minutes. Antibody combinations used were: CD45-PeCy7/CD271-APC/CD140a-PE, CD45-PeCy7/CD271-APC/CD140b-PE or an isotype controls combination (CD271-APC was from Miltenyi Biotec, Bergisch Gladbach, Germany, all other antibodies from BD Biosciences, Oxford, UK). The cells were washed and re-suspended in FACs buffer containing 100 ng/ml 4',6-diamidino-2-phenylindole (DAPI) before analysing on a BD LSRII flow cytometer. Dead cells were excluded from the analysis using DAPI before gating on the CD45-CD271+ population and assessing the expression of CD140a and CD140b (PDGF receptors α and β, respectively) on these cells.

### Cell counts and serum PDGF-AA and PDGF-BB measurements in PB

The total white cell count, platelet counts and CRP measurements were processed by the Leeds Teaching Hospitals patient diagnostics laboratory. For investigations involving patients’ sera, PB samples collected in clot accelerant tubes were allowed to stand for 30 minutes at room temperature (RT) prior to centrifugation for 15 minutes at 2000 g. The serum was aliquoted (1 ml/tube) and frozen at −80°C. The levels of two PDGF isoforms, −AA and –BB, were measured using commercially available enzyme-linked immunosorbent (ELISA) assay kits (Quantikine® ELISA kits, R&D Systems, Abingdon UK).

Both MSC colony counts and PDGF levels were analysed as the raw data (as CFU-F/ml or PDGF/ml), as well normalized to day 0 (the latter indicative of increase/decrease compared to the day 0 baseline, that is, <1 decrease, >1 increase). The healthy control group recruited for PDGF measurements (n =9) comprised five women and four men who were non post-traumatic healthy volunteers, with median ages of 35 (range 19 to 63 years) and 35 (range 22 to 63 years), respectively, for the female and male groups.

### MSC proliferation in response to patient’s serum

The effect of patient’s serum on MSC proliferation *in vitro* was assessed in a colorimetric cell proliferation assay based on a tetrazolium salt XTT (Roche, Welwyn Garden City, UK), as described previously [27]. In brief, the assay was performed using cultured MSCs from four donors, in quadruplicate for each cell seeding density and each serum sample. Cells were seeded at 125, 250 and 500 MSCs/well in 96-well plates and were allowed to attach for 24 hours in (D)MEM/2% FCS (both from Invitrogen, Paisley UK); the next day media were replaced with 150 μl of either non-haematopoietic (NH) media (positive control wells), (D)MEM/10% FCS not optimized for MSC growth (negative control wells) or (D)MEM/10% patient’s serum (test wells). MSCs were allowed to grow and the assay was stopped on day 5 by replacing the growth media with the XTT labelling mixture; the colour change was read at 450 and 620 nm using Opsys MR Plate reader (Dynex Technologies, Worthing, UK). Optical densities (ODs) were analysed separately for each seeding density and MSC culture and normalised to the OD of the positive control (NH media); the values for four MSC cultures were next averaged for each seeding density and serum proliferative indices (PIs <1 = less proliferative than NH, >1 = more proliferative than NH) were recorded for each time-point, for each serum.

### Statistical analysis

As a Gaussian distribution could not be assumed due to the sample size, non-parametric tests were carried out. The Mann–Whitney test was used to compare differences between two independent samples. The Wilcoxon signed-rank test was carried out to compare differences between paired samples. The Chi-square test was used for comparison of nominal data. Spearman’s rank correlation coefficient was used to test the relationship between two variables. A *P*-value of <0.05 was considered statistically significant and denoted by *. All statistical analysis was carried out using IBM SPSS statistics 19 and all graphical figures were made in Prism 6 (GraphPad Software, Inc.).

## Results

### MSC dynamics post fracture

**Figure 1:**
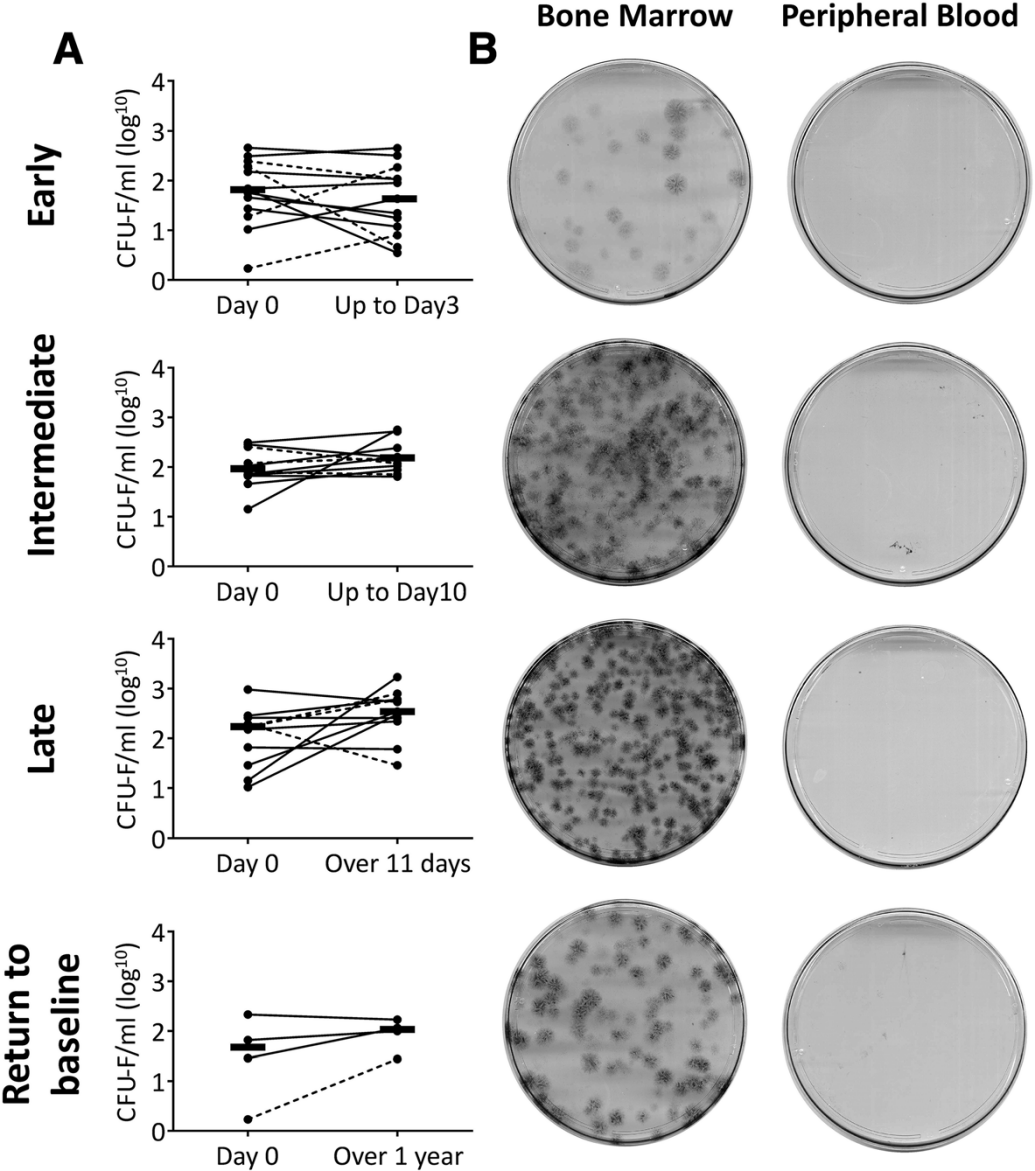
**MSC dynamics in patients’ bone marrow (BM) and peripheral blood (PB) over time post injury. A)** MSC changes in aspirated BM at different time-points post-injury (split into Subgroups: Early (Days 1 to 3 post injury), Intermediate (days 4 to 10 post injury), Late (more than 11 days post injury) and Return to baseline (more than 1 year post injury). The solid line represents polytrauma patients and the broken line represents isolated trauma patients; thick bars represent median values. **B)** Representative Day 0 CFU-F dishes from patients from the corresponding subgroups, demonstrating the abundance of CFU-Fs in the BM and their absence in PB. CFU-F, colony-forming unit – fibroblast; MSC, mesenchymal stem cell.

In total, 33 subjects were recruited into this study (Table 1). There were no statistically significant differences in the age and gender distributions between the two trauma and control groups. As expected, the differences in trauma severity (ISS) between the isolated trauma and polytrauma groups were statistically significant (Table 1). All patients had a sample (BM and PB) taken within 24 hours of injury (as baseline, Day 0) and a few days later, coinciding with the time when the patients were in the operating theatre for further surgical procedures (Figure 1). Subsequently, the data were split into four subgroups representing early response to fracture (1 to 3 days post injury), intermediate response (4 to 10 days), late response (11 or more days) and return to baseline (over 1 year). These time points were selected to represent the different phases of fracture healing [28].

Consistent with previous findings [22,29], large donor-to-donor variation (up to 100-fold, from 10 to 1,000 CFU-F/ml) was observed for both trauma groups (Figure 1A) and in the control group (median 140 CFU-F/ml, range 33 to 265, n =7). Some trends for increased MSCs per 1 ml of BM aspirate were observed in Intermediate and Late subgroups as well as in four patients studied long-term (not significant). IC MSC numbers or responses were not markedly different between the two injury severity groups (Figure 1A, solid line – polytrauma, broken line – isolated trauma).

Finally, there was no evidence for systemic mobilisation of MSCs in any of the groups (Figure 1B). Since no gross differences in MSC behaviours in isolated trauma and polytrauma groups were found, all subsequent analysis was performed on aggregated (pooled) data. Also BM MSC changes after fracture were relatively subtle in comparison with much larger donor-to-donor variation; therefore, all subsequent analyses were performed on data normalized to the ‘baseline’ (Day 0) values for each individual patient.

### Link between BM MSCs and serum levels of platelet-derived growth factors

**Figure 2:**
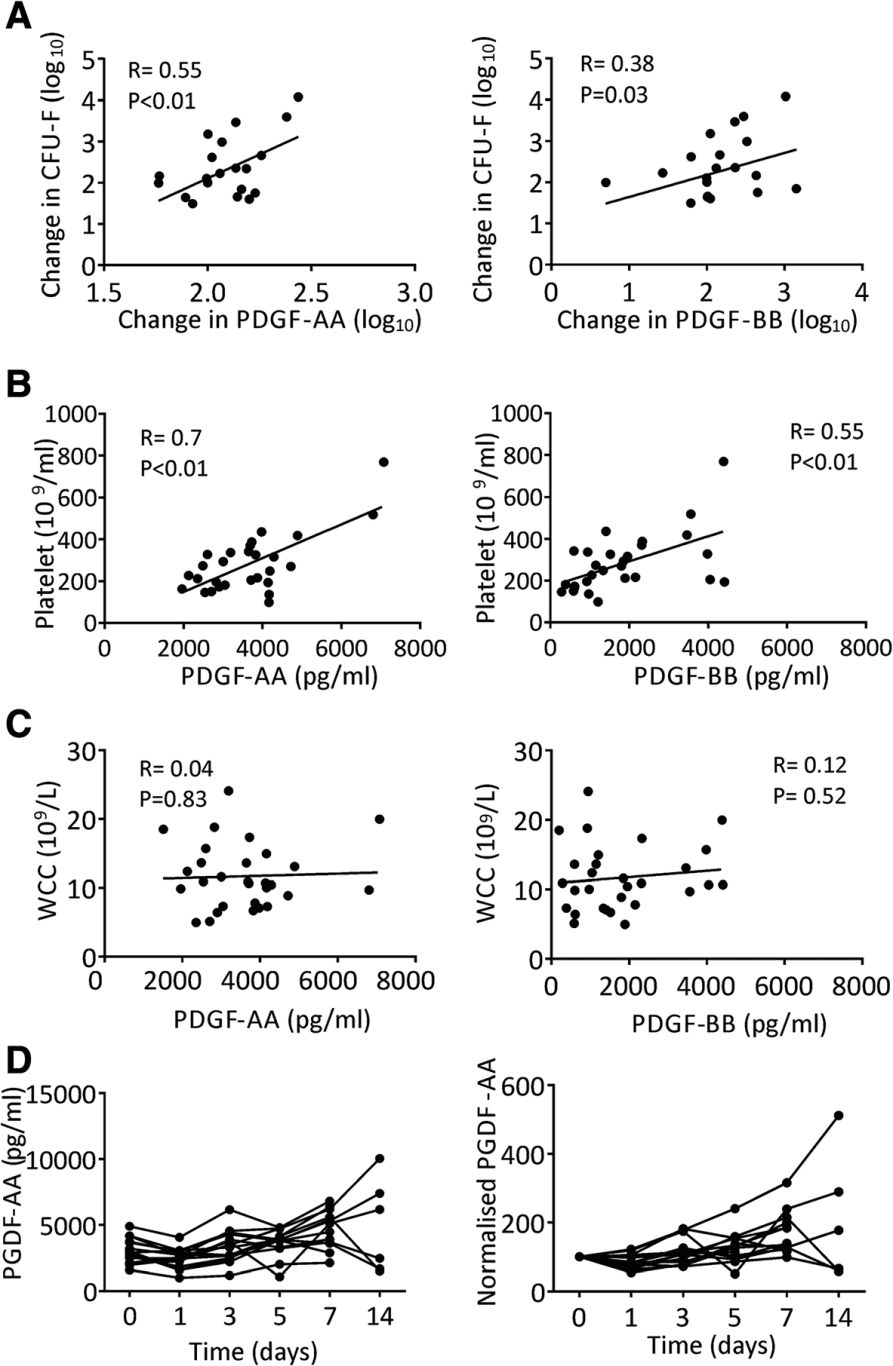
**Relationship between serum PDGF-AA and –BB and BM MSC numbers. A)** Correlations between changes in serum PDGF-AA or -BB with changes in BM MSCs in the same patient. Baseline (Day 0) data for each patient were denoted as 100% and the second time points are shown as a proportionate fold-change. **B)** Correlations between serum PDGF-AA or -BB and circulating platelet levels in the same patient. **C)** The lack of correlations between serum PDGF-AA or -BB with circulating white cell counts (WCC) in the same patients. **D)** Trends for PDGF-AA changes over time, with initial decline after trauma followed by a gradual rise by day 7 post trauma. Left panel – raw data, right panel – data normalised to baseline (Day 0 shown as 100%). BM, bone marrow; MSC, mesenchymal stem cell; PDGF, platelet-derived growth factor.

We next correlated individual changes in patients’ BM MSCs with time-matched changes in their serum PDGF-BB. As the role of PDGF-AA on MSC function is less-known, we also measured PDGF-AA in patient’s serum and correlated its changes after injury with that of MSCs (Figure 2). In these experiments, we also investigated individual changes in blood platelet counts in the same patient, as both PDGF-AA and-BB are known to be produced by activated platelets [30] and platelet increases have been described as a feature of the systemic inflammatory response after trauma including fractures [3]. Total white blood cells (WBCs) and CRP levels were additionally used to evaluate the systemic inflammatory response in these patients.

When both the cytokine levels and MSC levels were normalised to Day 0 levels for each individual patient (denoted as 100%), positive correlations were found between changes in BM MSCs and changes in both PDGF-AA and-BB (Figure 2A). The levels of both -AA and -BB significantly correlated with that of platelets (Figure 2B); no such correlations were found with WBC counts (Figure 2C) or with CRP [see Additional file 1: Figure S1]. This suggested that serum PDGF levels in post-trauma patients were influenced by levels of circulating platelets at that time rather than by a systemic inflammatory response.

Similarly to previously published data on PDGF-BB dynamics post-trauma [16], the levels of PDGF-AA showed an overall initial decline following trauma, before rising by an average of 1.7-fold by day 7 (Figure 2D). In approximately 20% of trauma patients, PDGF-BB and –AA levels had risen above the normal physiological levels on day 7; this was not the case for the control group, in which PDGF-AA and –BB remained within the normal physiological range [31] (median 4,128 pg/ml and 1,356 pg/ml, ranges 2,482 to 5,261 and 623 to 1,996, respectively, n =9). Similar to our BM MSC findings, no correlations were observed between the levels of PDGF-AA or -BB and ISS, or between isolated trauma and polytrauma groups [see Additional file 2: Figure S2].

The observed parallel changes in serum PDGFs in patients’ blood and the numbers of MSCs in their IC BM post-injury suggested a potential systemic effect of endogenous PDGFs on MSCs at the site distant to injury.

### Patients’ sera induce MSC proliferation

**Figure 3:**
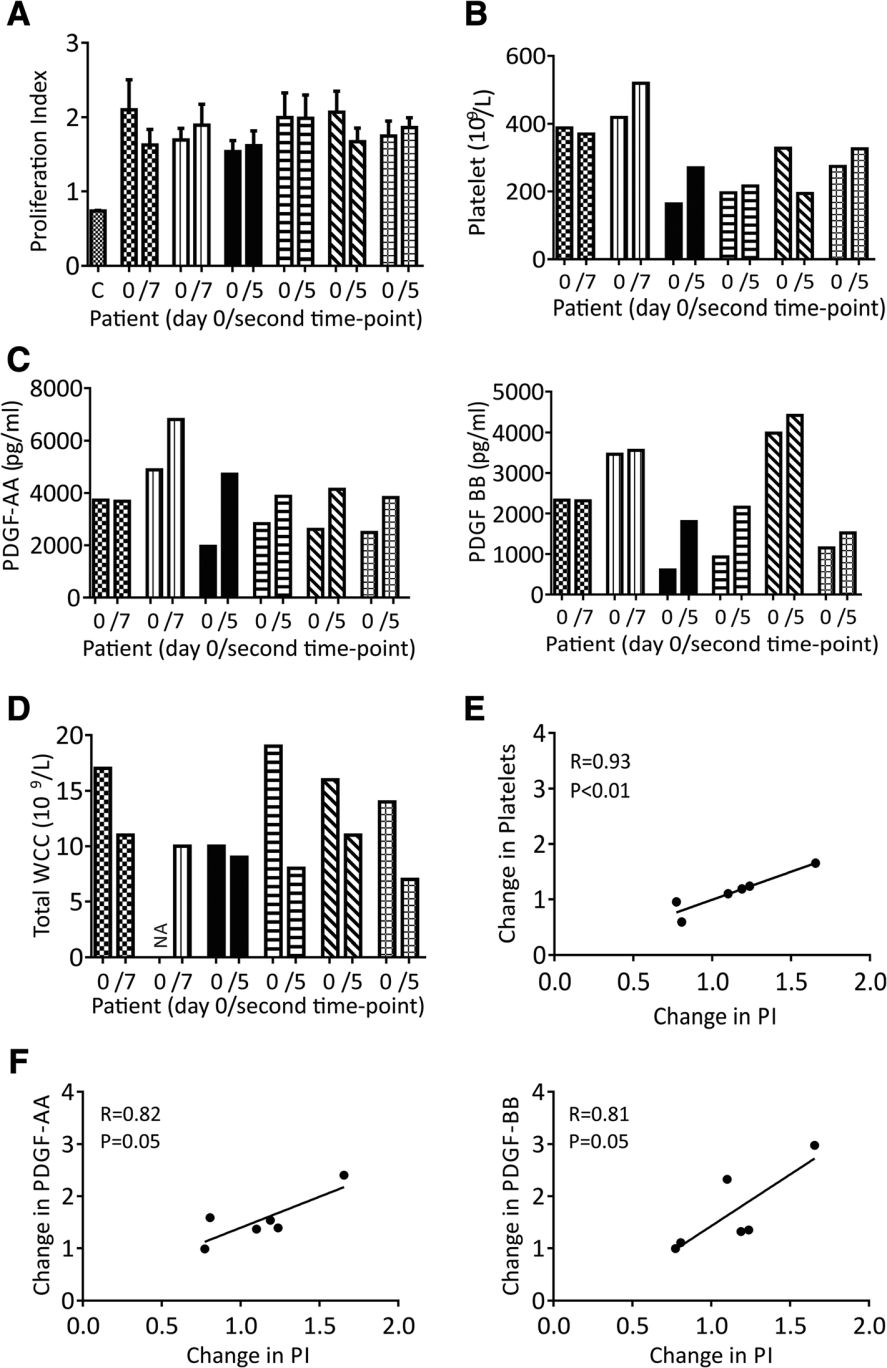
**The effects of serum PDGF-AA and –BB on MSC proliferation. A)** Proliferation indices (PIs), measured by xtt assay, of sera derived from six patients at two time-points; adjacent bars represent Day 0 Baseline and the second time point; C represents negative control FCS, error bars represent technical replicates. PIs were normalised to control NH media (PI =1). **B)** Platelet levels in PB of the same six patients taken at the same two time-points. **C)** PDGF-AA and -BB levels in PB of the same six patients taken at the same two time-points. **D)** White cell levels in PB of the same six patients taken at the same two time-points. Patterns in PI changes are similar to changes in PDGF-AA and -BB levels and platelet counts but not WCC counts. **E** and **F**) Positive correlations between changes in sera PIs and changes in the corresponding levels of PDGF-AA, −BB and platelets, in the same patient (<1 decline, >1 increase). MSC, mesenchymal stem cell; NH, non-haematopoietic; PB, peripheral blood; PDGF, platelet-derived growth factor; WCC, white cell count.

To further explore the link between changes in serum PDGFs and BM MSCs in the studied cohort of patients, we investigated whether patient’s serum had an effect on MSC proliferation in a PDGF-dependent manner (Figure 3). As expected, negative control medium produced very low proliferation indices (PIs) of 0.74, 0.72 and 0.72, for 125, 250 and 500 cells/well densities, respectively. Interestingly, patient’s sera had more ‘proliferation-inducing power’ than a positive control NH medium (PIs >1). The data for the 125 cells/well density are shown in Figure 3A. The other two seeding densities produced very similar results (not shown).

We next compared the patterns of individual changes in serum PIs with the corresponding changes in their PDGF and platelet levels (Figure 3, B-D) and very similar patterns of changes were found. In contrast, blood WCC patterns of change were drastically different to serum PI patterns (Figure 3E) emphasizing the specific contribution of platelet-derived factors to serum proliferative power. When the observed changes in serum PIs (for each patient) were correlated with the corresponding changes in their PDGF-AA, −BB or platelets, significant positive correlations were found (Figure 3E and F).

Such trends were not observed when raw PDGF-AA, −BB or raw platelet values were used, indicating that, similar to our findings with BM MSCs, the observed phenomena were patient-specific and that other serum molecules were most likely involved in controlling MSC proliferation. Because patients’ sera affected *in vitro* MSC proliferation in a PDGF-dependent manner, we concluded that a similar PDGF-driven effect on MSC proliferation could take place *in vivo*, thus explaining the observed increases in BM MSCs after injury in some patients.

### Patient’s MSCs express PDGF receptors and modulate their expression in response to changes in platelets

**Figure 4:**
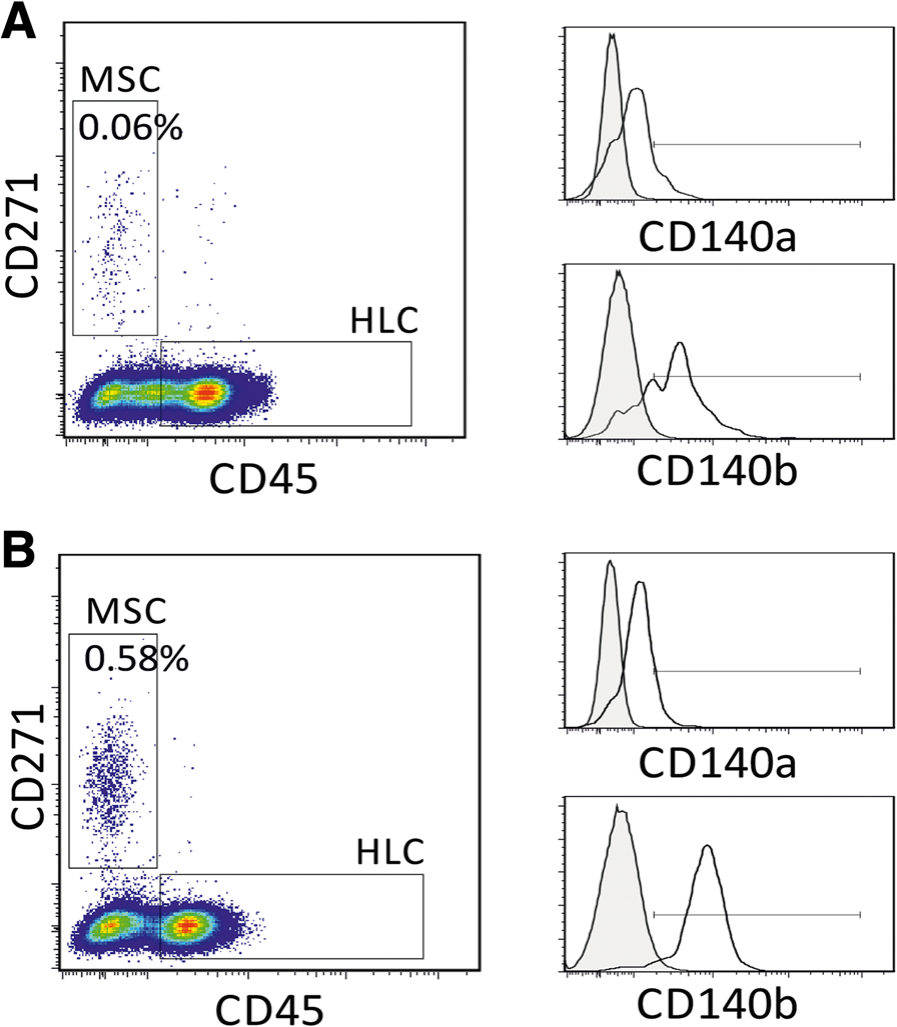
**Enumeration of BM MSCs (left panels) and PDGFR expression on MSCs (right panels) at the point of injury (Day 0 baseline, A) and five days later (B).** MSCs and control haematopoietic lineage cells (HLCs) are identified as CD45-CD271+ and CD45 + CD271- cells, respectively. Right panels - the expression of CD140a and CD140b on MSCs (empty histograms) and HLCs (shaded histograms); flow cytometry plots from a representative trauma patient are shown. BM, bone marrow; MSCs, mesenchymal stem cells; PDGFR.platelet-derived growth factor receptor.

To investigate other potential changes in BM MSCs in response to endogenous PDGFs, we investigated the expression of PDGF receptors α and β (CD140a and CD140b) on their surface [32] and analysed BM MSCs based on their native CD45-CD271+ phenotype, as previously reported [21,22,24,32,33]. Figure 4 shows the gating for MSCs and control haematopoietic lineage cells (HLCs) and the expression of CD140a and CD140b on both MSCs and HLCs from a representative trauma patient (Day 0 baseline – top panel, Day 5 – bottom panel).

**Figure 5:**
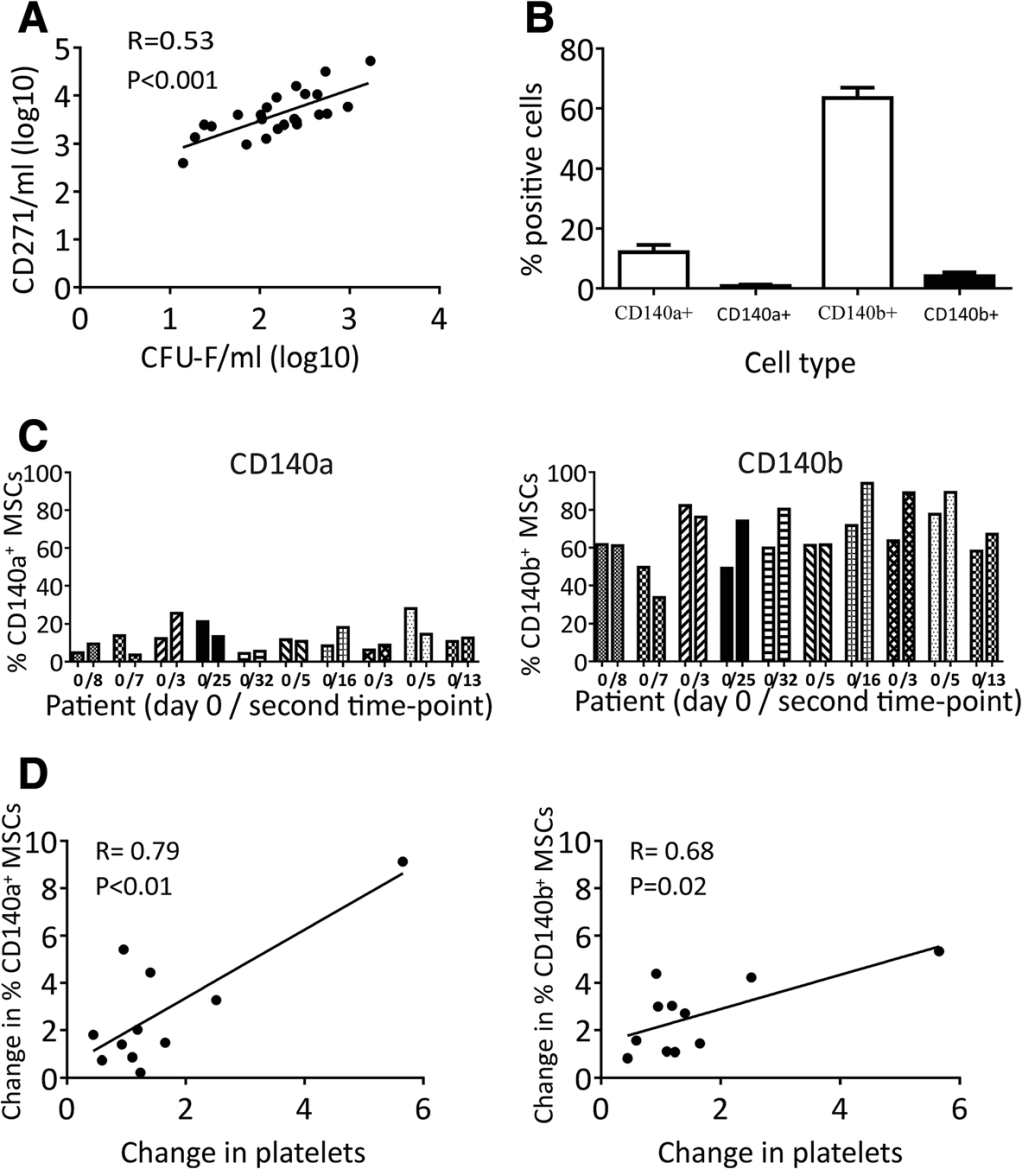
**PDGF receptor expression on BM MSCs, in relation to systemic changes, by flow cytometry. A)** Positive correlation between BM MSCs quantified by CFU-Fs assay and by flow cytometry for the CD45-CD271+ phenotype. **B)** Average proportions of PDGFRα(CD140a)- and PDGFRβ(CD140b)-positive subpopulations within BM MSCs (empty bars) and control HLCs (dark bars), showing the abundance of both PDGF receptors on MSCs compared to HLCs. Error bars represent SDs, n =10. **C)** CD140a (left panel) and CD140b (right panel) expression on CD45-CD271+ MSCs from 10 individual patients at two time-points; adjacent bars represent Day 0 Baseline and the second time point. **D)** Correlations between changes in proportion CD140a and CD140b receptor positive MSCs with changes in platelet levels in the same patient (<1 decline, >1 increase). BM, bone marrow; CFU-F, colony-forming unit-fibroblast; HLCs, haematopoietic lineage cells; MSCs, mesenchymal stem cells; PDGR, platelet-derived growth factor; SD, standard deviation.

Consistent with our previous findings [22], a significant correlation between BM MSC numbers measured by the CFU-F assay and by flow cytometry was evident (Figure 5A). CD45-CD271+ MSCs, but not HLCs, showed abundant PDGF receptors (Figure 5B). Interestingly, CD140a was expressed at lower levels on MSCs than CD140b (average 12% and 64% positive cells, respectively), consistent with previous studies on cultured MSCs [34] (Figure 5B). The minimal presence of both PDGF receptors on HLC indicated that PDGFs –AA and –BB in patients’ sera preferentially targeted MSCs rather than HLCs. In individual patients, percentages of CD140a- or CD140b-positive MSCs were not static and changed post-trauma showing no similarities with each other (Figure 5C). When changes in the percentages of CD140a- and CD140b-positive MSCs were correlated with time-matched changes in patients’ blood (in the same patient), the strongest correlations were observed with platelets themselves (Figure 5D).

## Discussion

In this study we investigated *in vivo* MSC responses at sites remote from acute skeletal trauma and related these to the systemic responses associated with fracture. We observed no major changes in aspirated MSC numbers following fracture; however, we were able to detect more subtle, patient-specific changes that were related to time-matched changes in the concentrations of PDGF–AA and –BB in patients’ sera with these cytokines, in turn, correlating with changes in platelet numbers. Both changes in MSCs and PDGFs were mostly within the normal physiological ranges indicating that the observed link between MSCs and PDGFs may be a normal physiological phenomenon rather than an injury-related one. The expression of PDGF receptors CD140a and CD140b on MSCs *in vivo* and the PDGF-depended effect of patient serum on MSC proliferation *in vitro* confirmed the biological plausibility of our observations. Conversely, we did not observe MSC mobilisation into the blood, even in the most severe trauma cases. Correspondingly, over a follow up period of up to two years, none of the patients were observed to develop any heterotopic ossifications, indicative of MSC entrapment in tissues. Systemic MSC release, their circulation and preferential homing to the sites of fracture has been previously documented in animal models [12,35]. Our data show that if this happens after skeletal injury in humans, the time frame for such release must be exceptionally short-lived, as circulating MSCs were not detectable in patients’ PB within a few hours post-injury. It is, therefore, plausible that any released MSCs could end up trapped in the lungs [36]. This finding further highlights the existence of potentially critical differences in the MSC biology between animal and human species [37].

To the best of our knowledge, only one previous study described changes in BM MSC numbers in fracture patients; however, the timing post-trauma was not reported [38] and no correlations with any systemic factors were made. More recently, Marsell *et al*. showed an approximately 50% increase in CD73+ MSCs in the BM and contralateral limbs of injured mice 7 to 14 days post intramedullary injury; the authors linked the mobilisation of MSCs to the systemic induction of bone morphogenetic protein 2 (BMP2} [39]. In our study, rather than BMP2, we measured two platelet-derived growth factors – PDGF-AA and -BB in patients’ sera, as well as their corresponding receptors – CD140a and CD140b – on the surface of CD45-CD271+ MSCs, from the same patient and at the same time-matched point post-injury. We chose PDGF-BB based on a large body of literature indicating that PDGF-BB was a major cytokine responsible for MSC proliferation [14,18,19,40,41]. PDGFB and A genes have been also shown to be expressed locally at healing sites between one to six weeks following injury [42]. Additionally, PDGF-AA and its receptor CD140a were selected because of recent publications indicating CD140a-positivity of ‘true’ *in vivo* MSCs in a mouse system [43,44].

To address a possible mechanism behind the systemic effect on platelet-derived factors on patient’s BM MSCs *in vivo*, we next investigated the effects of patients’ sera on MSC proliferation *in vitro.* As expected, the serum pairs with the highest rises in platelets (and PDGFs) had the highest rises in their proliferative indices. Therefore, it could be suggested that platelet-derived factors similarly induced MSC proliferation *in vivo*. It is possible that changes in platelet levels in our cohort of patients represented their bodies’ responses to the loss of platelets due to bleeding/consumption, affecting their increased production in order to reach normal physiological levels. Such recovery of platelets (average platelet half-life of 100 hours [45]) and associated increases in PDGFs, could, therefore, be responsible for parallel increases in MSCs in some patients. MSCs responded to endogenous PDGF-driven stimulation by changing the expression of PDGF receptors CD140a and CD140b on their surface. Physiological fluctuations in CD140a expression on the surface of human MSCs may, therefore, limit its utility as a specific MSC marker in humans [44].

## Conclusions

Platelet lysates or autologous human serum rich in PDGFs and other cytokines and chemokine molecules are currently used for *ex vivo* MSC expansion [34,41,46]. Similarly, platelet-rich plasma (PRP) is often used clinically to facilitate healing in tendons, cartilage and other skeletal tissues [47-49]. In this context, our study provides the first evidence that platelet-derived products could systemically influence physiological MSC behaviours in humans and that these MSC responses may be relatively fast. In this respect, our data provide a biological explanation for boosted MSC responses following PRP injections into the iliac crest of human volunteers, documented recently [50]. Because the observed correlations were valid in respect to paired changes in platelets and PDGFs (that is, for individual patients) and not for absolute values of PDGF-AA or -BB/ml (that is, for the whole patient cohort), our data also indicate that other systemic modulators are additionally involved in the systemic control of the BM MSC pool in humans. With a further proteomics study of patients’ sera, these other systemic factors could be identified and potentially tested in the future for their ability to ‘reactivate’ dormant MSCs lodged in non-healing tissues [51]. Therefore, we believe that our study contributes to a better understanding of human MSC biology *in vivo* and could potentially lead to the development of novel ways of systemically targeting endogenous MSCs in a variety of diseases.

## Additional files

Additional file 1: Figure S1. The lack of correlation between the levels of time-matched PDGF-AA (A) and PDGF-BB (B) and CRP. Additional file 2: Figure S2. The lack of correlation between the levels of PDGF-AA (A) and PDGF-BB (B) and ISS, or between isolated trauma group (triangles) and polytrauma group (diamonds) at baseline (Day 0).

## Abbreviations

BM: bone marrow
BMP: bone morphogenetic protein
BSA: bovine serum albumin
CFU-F: colony forming unit fibroblast
CRP: C reactive protein
(D)MEM: (Dulbecco’s) modified Eagle's medium
EDTA: ethylenediaminetetraacetic acid
FCS: fetal calf serum
HLC: haematopoietic lineage cells
IC: iliac crest
ISS: injury severity score
MNC: mononuclear cell
MSCs: mesenchymal stem cells
NH: non haematopoietic
OD: optical densities
PB: peripheral blood
PBS: phosphate-buffered saline
PDGF: platelet-derived growth factor
PI: proliferation indices
PRP: platelet rich plasma
WBC: white blood cell

## References

[1] Gerstenfeld LC, Cullinane DM, Barnes GL, Graves DT, Einhorn TA (2003). Fracture healing as a post-natal developmental process: molecular, spatial, and temporal aspects of its regulation. J Cell Biochem.

[2] Keel M, Trentz O (2005). Pathophysiology of polytrauma. Injury.

[3] Kumar PJ, Clark ML (2009). Kumar & Clark's Clinical Medicine.

[4] Hensler T, Sauerland S, Bouillon B, Raum M, Rixen D, Helling HJ, Andermahr J, Neugebauer EA (2002). Association between injury pattern of patients with multiple injuries and circulating levels of soluble tumor necrosis factor receptors, interleukin-6 and interleukin-10, and polymorphonuclear neutrophil elastase. J Trauma.

[5] Gebhard F, Pfetsch H, Steinbach G, Strecker W, Kinzl L, Bruckner UB (2000). Is interleukin 6 an early marker of injury severity following major trauma in humans?. Arch Surg.

[6] Marsell R, Einhorn TA (2011). The biology of fracture healing. Injury.

[7] Granero-Molto F, Weis JA, Miga MI, Landis B, Myers TJ, O'Rear L, Longobardi L, Jansen ED, Mortlock DP, Spagnoli A (2009). Regenerative effects of transplanted mesenchymal stem cells in fracture healing. Stem Cells.

[8] Ito H (2011). Chemokines in mesenchymal stem cell therapy for bone repair: a novel concept of recruiting mesenchymal stem cells and the possible cell sources. Mod Rheumatol.

[9] Mansilla E, Marin GH, Drago H, Sturla F, Salas E, Gardiner C, Bossi S, Lamonega R, Guzman A, Nunez A, Gil MA, Piccinelli G, Ibar R, Soratti C (2006). Bloodstream cells phenotypically identical to human mesenchymal bone marrow stem cells circulate in large amounts under the influence of acute large skin damage: new evidence for their use in regenerative medicine. Transplant Proc.

[10] Shyu WC, Lee YJ, Liu DD, Lin SZ, Li H (2006). Homing genes, cell therapy and stroke. Front Biosci.

[11] Ramirez M, Lucia A, Gomez-Gallego F, Esteve-Lanao J, Perez-Martinez A, Foster C, Andreu AL, Martin MA, Madero L, Arenas J, García-Castro J (2006). Mobilisation of mesenchymal cells into blood in response to skeletal muscle injury. Br J Sports Med.

[12] Kuznetsov SA, Mankani MH, Gronthos S, Satomura K, Bianco P, Robey PG (2001). Circulating skeletal stem cells. J Cell Biol.

[13] Jones E, McGonagle D (2008). Human bone marrow mesenchymal stem cells in vivo. Rheumatology (Oxford).

[14] Caplan AI, Correa D (2011). PDGF in bone formation and regeneration: new insights into a novel mechanism involving MSCs. J Orthop Res.

[15] Hollinger JO, Hart CE, Hirsch SN, Lynch S, Friedlaender GE (2008). Recombinant human platelet-derived growth factor: biology and clinical applications. J Bone Joint Surg Am.

[16] Pountos I, Georgouli T, Henshaw K, Bird H, Giannoudis PV (2013). Release of growth factors and the effect of age, sex, and severity of injury after long bone fracture. Acta Orthop.

[17] Gronthos S, Simmons PJ (1995). The growth factor requirements of STRO-1-positive human bone marrow stromal precursors under serum-deprived conditions in vitro. Blood.

[18] Gharibi B, Hughes FJ (2012). Effects of medium supplements on proliferation, differentiation potential, and in vitro expansion of mesenchymal stem cells. Stem Cells Transl Med.

[19] Pountos I, Georgouli T, Henshaw K, Bird H, Jones E, Giannoudis PV (2010). The effect of bone morphogenetic protein-2, bone morphogenetic protein-7, parathyroid hormone, and platelet-derived growth factor on the proliferation and osteogenic differentiation of mesenchymal stem cells derived from osteoporotic bone. J Orthop Trauma.

[20] Jones EA, Kinsey SE, English A, Jones RA, Straszynski L, Meredith DM, Markham AF, Jack A, Emery P, McGonagle D (2002). Isolation and characterization of bone marrow multipotential mesenchymal progenitor cells. Arthritis Rheum.

[21] Tormin A, Li O, Brune JC, Walsh S, Schutz B, Ehinger M, Ditzel N, Kassem M, Scheding S (2011). CD146 expression on primary nonhematopoietic bone marrow stem cells is correlated with in situ localization. Blood.

[22] Cuthbert R, Boxall SA, Tan HB, Giannoudis PV, McGonagle D, Jones E (2012). Single-platform quality control assay to quantify multipotential stromal cells in bone marrow aspirates prior to bulk manufacture or direct therapeutic use. Cytotherapy.

[23] Baker SP, O'Neill B, Haddon W, Long WB (1974). The injury severity score: a method for describing patients with multiple injuries and evaluating emergency care. J Trauma.

[24] Cox G, Boxall SA, Giannoudis PV, Buckley CT, Roshdy T, Churchman SM, McGonagle D, Jones E (2012). High abundance of CD271(+) multipotential stromal cells (MSCs) in intramedullary cavities of long bones. Bone.

[25] Hernigou P, Homma Y, Flouzat Lachaniette CH, Poignard A, Allain J, Chevallier N, Rouard H (2013). Benefits of small volume and small syringe for bone marrow aspirations of mesenchymal stem cells. Int Orthop.

[26] Churchman SM, Ponchel F, Boxall SA, Cuthbert R, Kouroupis D, Roshdy T, Giannoudis PV, Emery P, McGonagle D, Jones EA (2012). Transcriptional profile of native CD271+ multipotential stromal cells: evidence for multiple fates, with prominent osteogenic and Wnt pathway signaling activity. Arthritis Rheum.

[27] Jones EA, Crawford A, English A, Henshaw K, Mundy J, Corscadden D, Chapman T, Emery P, Hatton P, McGonagle D (2008). Synovial fluid mesenchymal stem cells in health and early osteoarthritis: detection and functional evaluation at the single-cell level. Arthritis Rheum.

[28] Einhorn TA (2005). The science of fracture healing. J Orthop Trauma.

[29] Veyrat-Masson R, Boiret-Dupre N, Rapatel C, Descamps S, Guillouard L, Guerin JJ, Pigeon P, Boisgard S, Chassagne J, Berger MG (2007). Mesenchymal content of fresh bone marrow: a proposed quality control method for cell therapy. Br J Haematol.

[30] Hart CE, Bailey M, Curtis DA, Osborn S, Raines E, Ross R, Forstrom JW (1990). Purification of PDGF-AB and PDGF-BB from human platelet extracts and identification of all three PDGF dimers in human platelets. Biochemistry.

[31] Madsen CV, Steffensen KD, Olsen DA, Waldstrom M, Smerdel M, Adimi P, Brandslund I, Jakobsen A (2012). Serial measurements of serum PDGF-AA, PDGF-BB, FGF2, and VEGF in multiresistant ovarian cancer patients treated with bevacizumab. J Ovarian Res.

[32] Buhring HJ, Battula VL, Treml S, Schewe B, Kanz L, Vogel W (2007). Novel markers for the prospective isolation of human MSC. Ann N Y Acad Sci.

[33] Maijenburg MW, Kleijer M, Vermeul K, Mul EP, van Alphen FP, van der Schoot CE, Voermans C (2012). The composition of the mesenchymal stromal cell compartment in human bone marrow changes during development and aging. Haematologica.

[34] Siegel G, Kluba T, Hermanutz-Klein U, Bieback K, Northoff H, Schafer R (2013). Phenotype, donor age and gender affect function of human bone marrow-derived mesenchymal stromal cells. BMC Med.

[35] Kitaori T, Ito H, Schwarz EM, Tsutsumi R, Yoshitomi H, Oishi S, Nakano M, Fujii N, Nagasawa T, Nakamura T (2009). Stromal cell-derived factor 1/CXCR4 signaling is critical for the recruitment of mesenchymal stem cells to the fracture site during skeletal repair in a mouse model. Arthritis Rheum.

[36] Nystedt J, Anderson H, Tikkanen J, Pietila M, Hirvonen T, Takalo R, Heiskanen A, Satomaa T, Natunen S, Lehtonen S, Hakkarainen T, Korhonen M, Laitinen S, Valmu L, Lehenkari P (2013). Cell surface structures influence lung clearance rate of systemically infused mesenchymal stromal cells. Stem Cells.

[37] Kuznetsov SA, Mankani MH, Leet AI, Ziran N, Gronthos S, Robey PG (2007). Circulating connective tissue precursors: extreme rarity in humans and chondrogenic potential in guinea pigs. Stem Cells.

[38] Seebach C, Henrich D, Tewksbury R, Wilhelm K, Marzi I (2007). Number and proliferative capacity of human mesenchymal stem cells are modulated positively in multiple trauma patients and negatively in atrophic nonunions. Calcif Tissue Int.

[39] Marsell R, Steen B, Bais MV, Mortlock DP, Einhorn TA, Gerstenfeld LC (2014). Skeletal trauma generates systemic BMP2 activation that is temporally related to the mobilization of CD73+ cells. J Orthop Res.

[40] Ng F, Boucher S, Koh S, Sastry KS, Chase L, Lakshmipathy U, Choong C, Yang Z, Vemuri MC, Rao MS, Tanavde V (2008). PDGF, TGF-beta, and FGF signaling is important for differentiation and growth of mesenchymal stem cells (MSCs): transcriptional profiling can identify markers and signaling pathways important in differentiation of MSCs into adipogenic, chondrogenic, and osteogenic lineages. Blood.

[41] Fekete N, Gadelorge M, Furst D, Maurer C, Dausend J, Fleury-Cappellesso S, Mailander V, Lotfi R, Ignatius A, Sensebe L, Bourin P, Schrezenmeier H, Rojewski MT (2012). Platelet lysate from whole blood-derived pooled platelet concentrates and apheresis-derived platelet concentrates for the isolation and expansion of human bone marrow mesenchymal stromal cells: production process, content and identification of active components. Cytotherapy.

[42] Andrew JG, Hoyland JA, Freemont AJ, Marsh DR (1995). Platelet-derived growth factor expression in normally healing human fractures. Bone.

[43] Morikawa S, Mabuchi Y, Kubota Y, Nagai Y, Niibe K, Hiratsu E, Suzuki S, Miyauchi-Hara C, Nagoshi N, Sunabori T, Shimmura S, Miyawaki A, Nakagawa T, Suda T, Okano H, Matsuzaki Y (2009). Prospective identification, isolation, and systemic transplantation of multipotent mesenchymal stem cells in murine bone marrow. J Exp Med.

[44] Pinho S, Lacombe J, Hanoun M, Mizoguchi T, Bruns I, Kunisaki Y, Frenette PS (2013). PDGFRalpha and CD51 mark human nestin + sphere-forming mesenchymal stem cells capable of hematopoietic progenitor cell expansion. J Exp Med.

[45] Sinzinger H, Fitscha P, Peskar BA (1986). Platelet half-life, plasma thromboxane B2 and circulating endothelial-cells in peripheral vascular disease. Angiology.

[46] Rubio-Azpeitia E, Andia I (2014). Partnership between platelet-rich plasma and mesenchymal stem cells: in vitro experience. Muscles Ligaments Tendons J.

[47] Wang JH (2014). Can PRP effectively treat injured tendons?. Muscles Ligaments Tendons J.

[48] Xie A, Nie L, Shen G, Cui Z, Xu P, Ge H, Tan Q (2014). The application of autologous plateletrich plasma gel in cartilage regeneration. Mol Med Rep.

[49] El Backly RM, Zaky SH, Muraglia A, Tonachini L, Brun F, Canciani B, Chiapale D, Santolini F, Cancedda R, Mastrogiacomo M (2013). A platelet-rich plasma-based membrane as a periosteal substitute with enhanced osteogenic and angiogenic properties: a new concept for bone repair. Tissue Eng Part A.

[50] Philippart P, Meuleman N, Stamatopoulos B, Najar M, Pieters K, De Bruyn C, Bron D, Lagneaux L (2014). In vivo production of mesenchymal stromal cells after injection of autologous platelet-rich plasma activated by recombinant human soluble tissue factor in the bone marrow of healthy volunteers. Tissue Eng Part A.

[51] Bajada S, Marshall MJ, Wright KT, Richardson JB, Johnson WE (2009). Decreased osteogenesis, increased cell senescence and elevated Dickkopf-1 secretion in human fracture non union stromal cells. Bone.

