# Supplementary figures and images for: The systemic influence of platelet-derived growth factors on bone marrow mesenchymal stem cells in fracture patients
Source: BMC Med. 2015 Jan 13;13:6. doi: 10.1186/s12916-014-0202-6 (PMC4293103; doi:10.1186/s12916-014-0202-6)

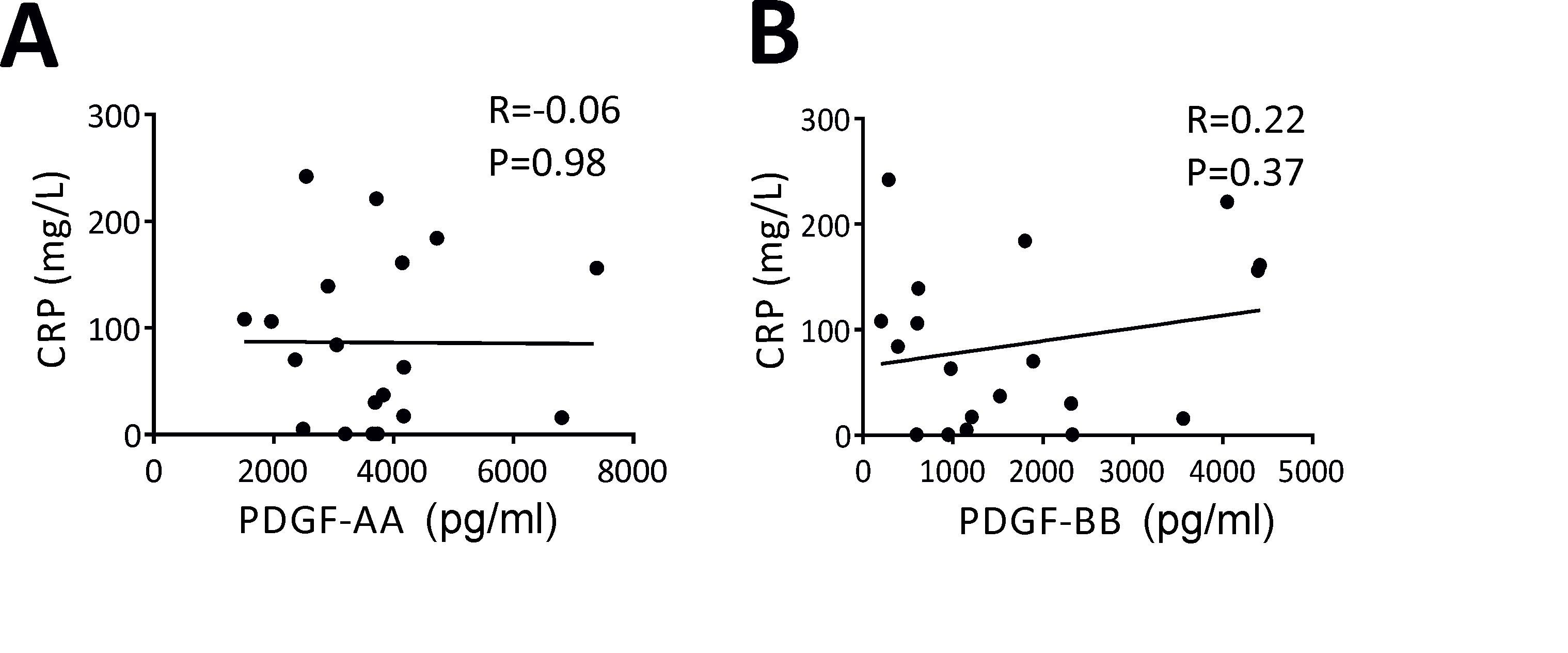

Supplement: Additional file 1: Figure S1. — The lack of correlation between the levels of time-matched PDGF-AA (A) and PDGF-BB (B) and CRP. [file 12916_2014_202_MOESM1_ESM.tiff]

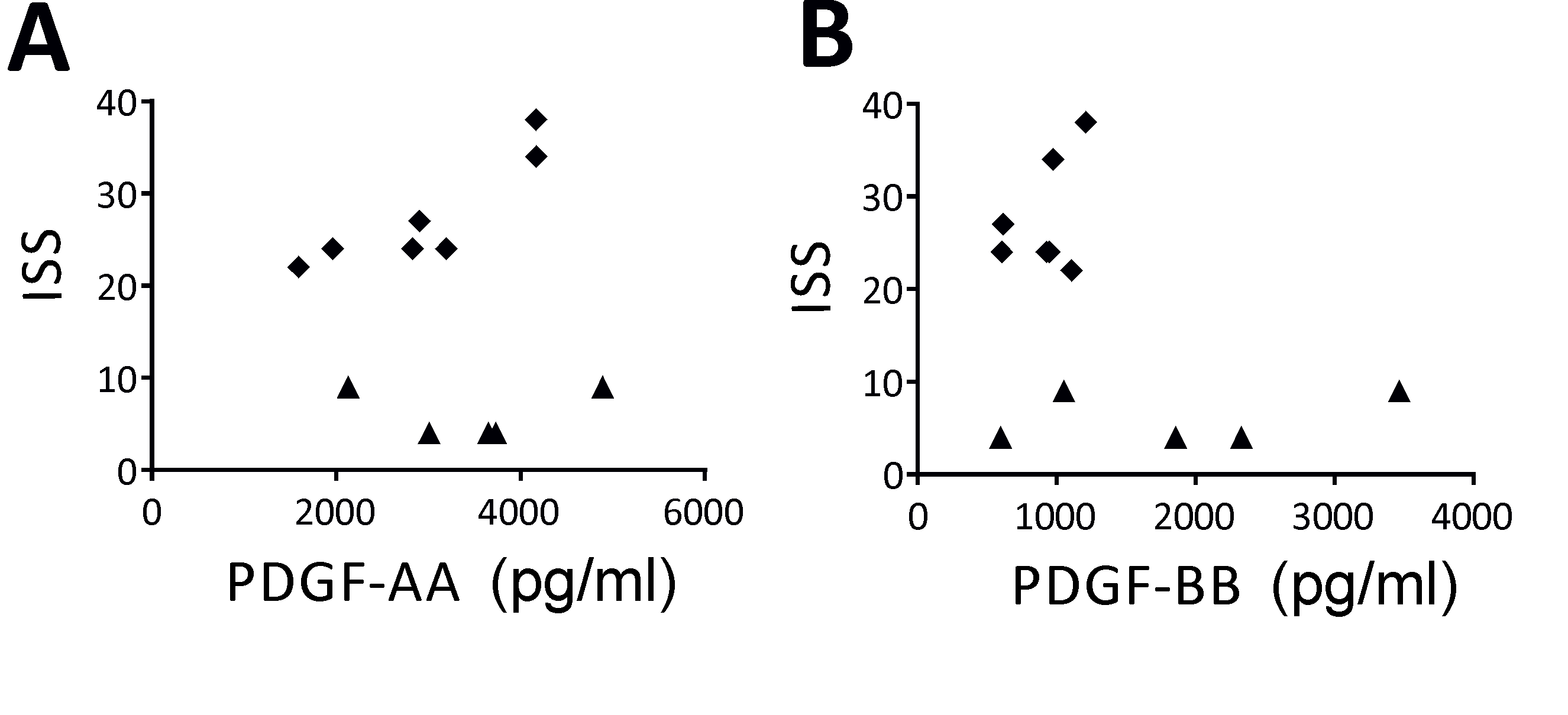

Supplement: Additional file 2: Figure S2. — The lack of correlation between the levels of PDGF-AA (A) and PDGF-BB (B) and ISS, or between isolated trauma group (triangles) and polytrauma group (diamonds) at baseline (Day 0). [file 12916_2014_202_MOESM2_ESM.tiff]
